# Pushing the Ligand Efficiency Metrics: Relative Group Contribution (RGC) Model as a Helpful Strategy to Promote a Fragment “Rescue” Effect

**DOI:** 10.3389/fchem.2019.00564

**Published:** 2019-08-16

**Authors:** Andrés Felipe Vásquez, Andrés González Barrios

**Affiliations:** ^1^Grupo de Diseño de Productos y Procesos (GDPP), School of Chemical Engineering, Universidad de los Andes, Bogotá, Colombia; ^2^Laboratorio de Fisiología Molecular, Instituto Nacional de Salud, Bogotá, Colombia

**Keywords:** ligand efficiency metrics, fragment-based screening, property-based design, drug discovery, protein-ligand interactions, structure-activity relationship, fragment library

## Abstract

The ligand efficiency (LE) indexes have long been used as decision-making criteria in drug discovery and development. However, in the context of fragment-based drug design (FBDD), these metrics often exhibit a strong emphasis toward the selection of highly efficient “core” fragments for potential optimization, which are not usually considered as parts of a larger molecule with a size typical for a drug. In this study, we present a relative group contribution (RGC) model intended to predict the efficiency of a drug-sized compound in terms of its component fragments. This model could be useful not only in rapidly predicting all the possible combinations of promising fragments from an earlier hit discovery stage, but also in enabling a relatively low-LE fragment to become part of a drug-sized compound as long as it is “rescued” by other high-LE fragments.

## Introduction

Ligand efficiency metrics have been applied as a decision-making strategy nearly universally accepted during the last two decades (Hopkins et al., 2014). They are intended to compare the quality of hits and leads during hit to lead and lead optimization phases of drug discovery (Cavalluzzi et al., 2017). Among these metrics, the ligand efficiency (LE), firstly described by Hopkins et al. (2004), continues to be the most widely used index to discriminate between promising molecules and those which are not (Reynolds, 2015). LE is defined by the following formula:

(1) $$\begin{array}{r} LE=\;\frac{\Delta G}{N} \end{array}$$

where ΔG = –RTInK_d_, N (also known as HAC or heavy atom count) represents the number of heavy, non-hydrogen atoms, and K_d_ corresponds to the equilibrium dissociation constant.

As shown in Equation (1), LE normalizes the potency by size, specifically representing the average contribution ΔG (Gibbs free energy) per heavy atom. LE is typically used in FBDD as cut-off criterion to retrieve just high-LE fragments in a screening process (Murray and Verdonk, 2006; Schultes et al., 2010). Intriguingly, because LE usually considers fragments as independent chemical entities, some other metrics have emerged to consider the change in affinity as a fragment is developed into a larger, high-affinity drug-sized compound. One prime example is the group efficiency (GE), described in 2008 (Verdonk and Rees, 2008), which allows measuring the contribution to the binding efficiency of a particular group of atoms added to an existing lead molecule:

(2) $$\begin{array}{r} \mathrm{GE}=\;\frac{\Delta \Delta G}{\Delta N} \end{array}$$

where ΔΔG = ΔG(B)-ΔG(A) and ΔN = (N(B)-N(A); in other words, ΔΔG is equal to the difference between the Gibbs free energy of the existing molecule (or fragment) “A” and the new combined molecule “B” and ΔN corresponds to the difference between the number of non-hydrogen atoms of molecules “A” and “B.” However, GE is based on a pairwise comparison of structurally closely related compounds and, hence, it is frequently applied for optimization of a high-efficient fragment (Hopkins et al., 2014). Therefore, a rapid and simple method for comparing efficiency of different fragments as part of a whole, including if they are dissimilar to each other (or if they occupy different pockets in the target molecule), needs to be developed.

Several independent studies in the past two decades have indicated an overemphasis on potency by the pharmaceutical industry (Albert et al., 2007; Hopkins et al., 2014). Still, other factors such as chemical novelty (Medina-Franco et al., 2014), selectivity fine-tuning (Costantino and Barlocco, 2018), structural alerts avoidance (Jasial et al., 2017), and synthetic accessibility (Fukunishi et al., 2014) are increasingly playing a key role in drug design. Considering these non-mutually exclusive events, we hypothesize that fragments that not necessarily exhibit a high efficiency level during a screening procedure, either virtual or experimental, would still have the possibility of taking part in a complete drug-sized compound.

In this study, we propose that a relative group contribution (RGC) model based on the efficiency of its component fragments may estimate the efficiency of a drug-sized compound. This model calculates the minimum efficiency required for unknown fragments by considering the efficiency of those already known, which facilitates a rapid elucidation of the best combinations of fragments. Likewise, this model facilitates that fragments with a relatively low efficiency may not necessarily be eliminated at an early stage of the screening process and, consequently, may become eventually represented as chemical moieties within the final candidate compound -a phenomenon herein referred to as fragment “rescue” effect.

## Theoretical Framework of the RGC Model

The proposed model is based on three main assumptions:

1. The efficiency of an entire molecule may be estimated as the weighted root mean square (WMRS) of the efficiency of its component fragments. The rational for using this type of mean is intended to (1) cope with the negative values of ΔG and (2) consider effectively the potentially different number of non-hydrogen atoms (N) for each component fragment.
2. The efficiency of each fragment (regarded as the entire ratio and not just the quotient) is, in principle, dependent on each other, excepting in cases of two fragments when the N for them is equal to each other (and then their weight is equivalent), or in cases when three or more fragments are involved.
3. The efficiency of each fragment is directly calculated from the ΔG resulting in its direct interaction with a specific location (i.e., binding site or pocket) in a particular receptor.

Our hypothesis assumes, according to its first principle, that the WRMS of the LEs of the fragments composing an entire molecule ($\bar{LE_{q}}$) corresponds to the actual LE of this latter (LE_T_) (A comprehensive list of mathematical terms is shown in Supplementary Material). Because this mean is intended to be proportionally similar to the real, total LE of an entire molecule (LE_T_), we refer herein to it as the apparent total LE (${\text{LE}}_{\text{T}}^{\text{app}}$):

(3) $$\begin{array}{r} {\bar{LE}}_{q}={LE}_{T}^{app}\approx \;{LE}_{T} \end{array}$$

For clarity of the RGC concept, we consider first the ${\text{LE}}_{\text{T}}^{\text{app}}$ as a simple arithmetic mean:

(4) $$\begin{array}{r} {LE}_{T}^{app}=\frac{1}{x}\left(LE_{1}+{LE}_{2}+{LE}_{3}+\ldots +{LE}_{x}\right) \end{array}$$

where LE_i_ corresponds to the LE of the component fragments (LE_1_, LE_2_, etc.), and x refers to the number of fragments composing the molecule. Therefore, once LE is expressed in terms of the Equation (1):

(5) $$\begin{array}{r} {LE}_{T}^{app}=\frac{1}{x}\left(\frac{\Delta G_{1}}{N_{1}}+\frac{\Delta G_{2}}{N_{2}}+\frac{\Delta G_{3}}{N_{3}}+\ldots +\frac{\Delta G_{x}}{N_{x}}\right)≅\;\frac{\Delta G_{T}}{xN_{T}} \end{array}$$

where ΔG_i_ is the change in Gibbs free energy for each composing fragment (ΔG_1_, ΔG_2_, etc.) up to a maximum number of fragments x and N_i_ correspond to the number of non-hydrogen atoms of each fragment. Similarly, ΔG_T_ and N_T_ represent the change in Gibbs free energy and number of non-hydrogen atoms for the entire molecule, respectively. Should be remembered that (5) is based on an arithmetic mean and hence it assumes that N is equal among all composing fragments, so that it would just be applicable in this specific scenario.

If we consider the Equation (5) for a molecule composed by a single fragment:

(6) $$\begin{array}{r} {LE}_{T}^{app}=\frac{1}{x}\;\overset{x}{\underset{i=1}{\sum }}{\left(\frac{\Delta G}{N}\right)}_{i}=\;{LE}_{1} \end{array}$$

we can observe that ${\text{LE}}_{\text{T}}^{\text{app}}$ correspond to the LE value of the unique component fragment, namely LE1, which supports a scenario where the component fragment is also the entire “final” molecule. However, expressing (5) for a molecule composed by two fragments:

(7) $$\begin{array}{r} {LE}_{T}^{app}=\frac{1}{x}\overset{x}{\underset{i=1}{\sum }}{\left(\frac{\Delta G}{N}\right)}_{i}=\frac{1}{2}\overset{2}{\underset{i=1}{\sum }}{\left(\frac{\Delta G}{N}\right)}_{i}=\;\frac{1}{2}\left({LE}_{1}+{LE}_{2}\right) \end{array}$$

we could notice that, in contrast to the one-fragment case, the existence of more than one compound allows for the solution of the equation in terms of a particular fragment:

(8) $$\begin{array}{r} {LE}_{2}=\;2{LE}_{T}^{app}-\;{LE}_{1} \end{array}$$

The last equation poses a simple but important principle: Starting from an “ideal” ${\text{LE}}_{\text{T}}^{\text{app}}$, a particular low-LE fragment can be successfully chosen or “rescued” by one or more high-LE fragments. Now, if we consider a three-fragment case:

(9) $$\begin{array}{r} {LE}_{T}^{app}=\frac{1}{x}\overset{x}{\underset{i=1}{\sum }}{\left(\frac{\Delta G}{N}\right)}_{i}=\frac{1}{3}\overset{3}{\underset{i=1}{\sum }}{\left(\frac{\Delta G}{N}\right)}_{i}=\;\frac{1}{3}\left({LE}_{1}+{LE}_{2}+{LE}_{3}\right) \end{array}$$

Interestingly, for this case, even if we assume in this example that we know LE_1_, it is still possible to consider a LE value for the unknown fragments LE_2_ and LE_3_ grouping them together into a single term:

(10) $$\begin{array}{r} {LE}_{T}^{app}=\frac{1}{3}\left({LE}_{1}+2{LE}_{\delta }\right) \end{array}$$

where the LE delta (LE_δ_) corresponds to a transient, “ideal” value intended to be equal for all the fragments which individual LE is still unknown. Therefore, as we will discuss below, this value will be modified as long as new LE values are known for fragments, independently of their position.

Likewise, assuming also that we just know LE_1_, we could express ${\text{LE}}_{\text{T}}^{\text{app}}$ for the two-fragment case:

(11) $$\begin{array}{r} {LE}_{T}^{app}=\frac{1}{2}\left({LE}_{1}+{LE}_{\delta }\right) \end{array}$$

which would indicate that:

(12) $$\begin{array}{r} {LE}_{\delta }={LE}_{2} \end{array}$$

This result suggests, as we also discuss below, that a LE_δ_ is expected to equal the LE value of a last fragment to be known (LE_u_), independently of the number of fragments and their position. This behavior corresponds to a subtractive average (SA). Remarkably, although the nature of LE_δ_ is somewhat similar to the cumulative average (CA) or moving average (MA), the number of fragments with unknown LE is continuously decreasing and LE_δ_ does not “run” within a predetermined window size.

Finally, assuming also that we just know LE_1_, we could express ${\text{LE}}_{\text{T}}^{\text{app}}$ for the one-fragment case:

(13) $$\begin{array}{r} {LE}_{T}^{app}=\left[\;{LE}_{1}+0\left({LE}_{\delta }\right)\right]={LE}_{1} \end{array}$$

indicating that LE_δ_ can only be calculated if there are at least two starting fragments and, more importantly, it is especially useful in cases of three or more of them.

At this point, think of the ${\text{LE}}_{\text{T}}^{\text{app}}$ as a whole for an undetermined series of fragments:

(14) $$\begin{array}{r} {LE}_{T}^{app}=\frac{1}{x}\left({LE}_{1}+\ldots {+LE}_{x}\right) \end{array}$$

If we assume again that we just know LE_1_, it is possible to rearrange ${\text{LE}}_{\text{T}}^{\text{app}}$ using LE_δ_:

(15) $$\begin{array}{r} {LE}_{T}^{app}=\frac{1}{x}\left[{LE}_{1}{\;+\left(x-1\right)LE}_{\delta }\right] \end{array}$$

where (x-1) corresponds to the coefficient of the LE_δ_ value independently of the number of starting fragments as shown in Equations (10, 11, 13). Hence, if we resolve for LE_δ_:

(16) $$\begin{array}{r} {LE}_{\delta }=\frac{1}{x-1}\left(x{{LE}_{T}^{app}\mathrm{- LE}}_{1}\right) \end{array}$$

Now, if we take into account that the number 1 in this equation actually corresponds to the number of known LE values of fragments or a, we can observe that:

(17) $$\begin{array}{r} if\;\left(x\text{ - }a\right)\to \;1,\;then\;LE_{\delta }\to \;LE_{u} \end{array}$$

What means that the more (x-a) tends to 1, the more LE_δ_ tends to LE_u_, just as we saw previously in Equation (12). Finally, considering the formula for LE_δ_ in terms of an undetermined number of fragments with different known and unknown LE values:

(18) $$\begin{array}{c} LE_{\delta }=\frac{1}{x-a}\left[\sum_{i=1}^{x}{\left(\frac{\Delta G}{N}\right)}_{i}-\;\sum_{j=0}^{a}{\left(\frac{\Delta G}{N}\right)}_{j}\right]\\ if\;and\;only\;if\;\{\begin{array}{l} 1\leq x<\infty \\ x\in \mathbb{N}\\ 0\leq a<x\\ a\in {\mathbb{Z}}_{0}^{+} \end{array} \end{array}$$

where the first summation term indicates the “ideal” sum of LE values for the existing fragments (LE_i_) as if they would have the same value, and the second summation term refers to the “real” sum of all fragments which LE value is already known (LE_j_). On the other hand, if a = o, LE_0_ would not proceed as a real value (and by extension the second summation term). Therefore, in this specific case a consequence would be that:

(19) $$\begin{array}{r} {{LE}_{\delta }=LE}_{T}^{app} \end{array}$$

Now, after having explained the basic concepts of RGC and LE_δ_, we could express ${\text{LE}}_{\text{T}}^{\text{app}}$ in terms of the WMRS according to our hypothesis:

(20) $$\begin{array}{r} {LE}_{T}^{app}=\sqrt{\overset{x}{\underset{i=1}{\sum }}{LE}_{i}^{2}w_{i}/\overset{x}{\underset{i=1}{\sum }}w_{i}}=\sqrt{\overset{x}{\underset{i=1}{\sum }}{\left(\frac{\Delta G}{N}\right)}_{i}^{2}w_{i}/\overset{x}{\underset{i=1}{\sum }}w_{i}} \end{array}$$

where LE_*i*_ corresponds to the LE of each component fragment, w_*i*_ refers to the weight of each fragment (depending on the N of each one) and x refers to the number of fragments.

Likewise, our formula for LE_δ_ would be:

(21) $$\begin{array}{c} LE_{\delta }=\frac{1}{\sqrt{w_{\delta }(x\text{ - }a)}}\left[\sqrt{\sum_{i=1}^{x}{\left(\frac{\Delta G}{N}\right)}_{i}^{2}w_{i}-\;\sum_{j=0}^{a}{\left(\frac{\Delta G}{N}\right)}_{j}^{2}w_{j}}\right]\\ if\;and\;only\;if\;\{\begin{array}{l} 1\leq x<\infty \\ x\in \mathbb{N}\\ 0\leq a<x\\ a\in {\mathbb{Z}}_{0}^{+} \end{array} \end{array}$$

The additional term in this equation, namely W_δ_, corresponds to the “ideal” weight of all fragments with unknown LE value, as if they would have the same value. Just as with LE_δ_, this parameter is expected to change with every new LE value of fragment known, until the value (weight) corresponding to the last fragment with unknown LE value is adopted.

## Fragment Selection and LE_T_ Prediction by the RGC Model

**Figure 1:**
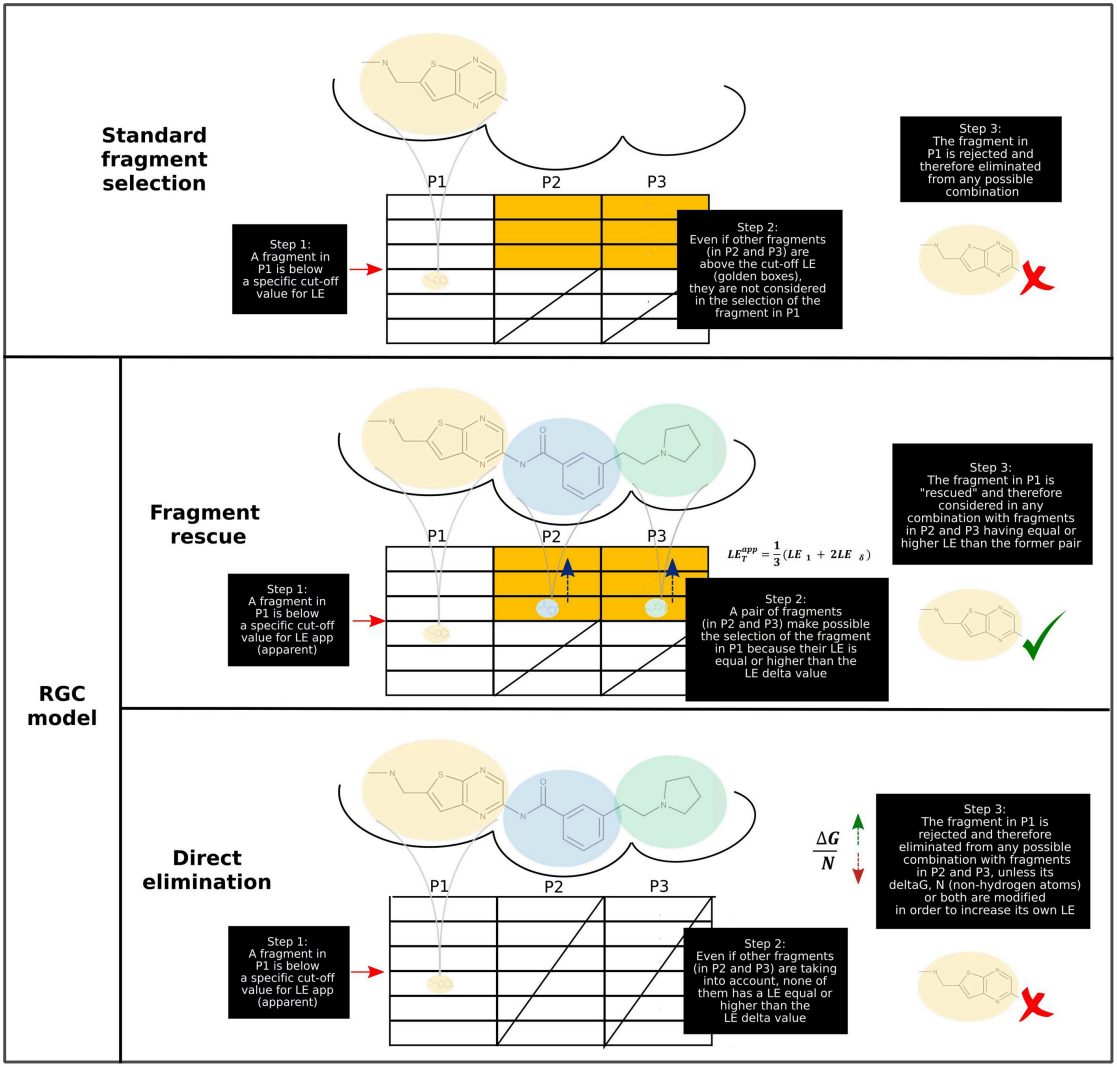
Application of RGC model to a hypothetical fragment-based drug design (FBDD) campaign. (**Upper**) In the “standard” or classical screening approach, a fragment is selected (i.e., can be part of a final drug-size compound) depending exclusively upon their own LE. If this parameter is not equal or greater than a pre-established cut-off value, the fragment is rejected. (**Lower**) According to the RGC model, a fragment is selected depending on the fragments on the other positions. Based on the presence of high-LE fragments in alternative positions (illustrated by yellow boxes), a low-LE fragment may become either rescued or rapidly discarded (using the dynamic LE_δ_ value in both cases).

As presented in a hypothetical example (Figure 1), three central premises may be elucidated for the RGC model by selecting hit compounds, starting from a cut-off LE value:

1. A low-efficiency fragment (i.e., with a LE < ${\text{LE}}_{\text{T}}^{\text{app}}$) can be rescued IF there exists high-efficiency fragments (i.e., with a LE > ${\text{LE}}_{\text{T}}^{\text{app}}$) in ALL the other positions (i.e., binding sites or pockets) of the target molecule, usually a protein. If this condition is not satisfied, the fragment could be automatically rejected from the set of possible combinations of fragments for an entire molecule.
2. If there are no high-efficiency fragments in all the other positions simultaneously, a molecular fragment with low-efficiency fragment can still be rescued IF at least one fragment in any other position with an efficiency high enough to reach exists, in average with the first, the ${\text{LE}}_{\text{T}}^{\text{app}}$. This implies, therefore, that once a fragment is rescued by one or more high-efficiency fragments in other positions, any fragment in subsequent unexplored positions is just required to have a mid-efficiency (with a LE ≅ LE_δ_ ideal) as a minimum. Additionally, each time LE_δ_ changes, a differential “pushing” over the LE of unknown fragments occurs in terms of ΔG and/or N, which could be potentially modified to achieve an acceptable LE value and could therefore be selected.
3. Even if a low-efficiency fragment is not rescued after implementing the strategies stated in Equation (1, 2), a potential rescue could still take place exploring a more diverse library sample of fragments, assuming that (1) you are dealing with a number of fragments well-below under the maximum theoretical chemical space for them (a population of about 10^7^ molecules) and (2) the fragment is not an outlier compared to other fragments intended to combine.

**Figure 2:**
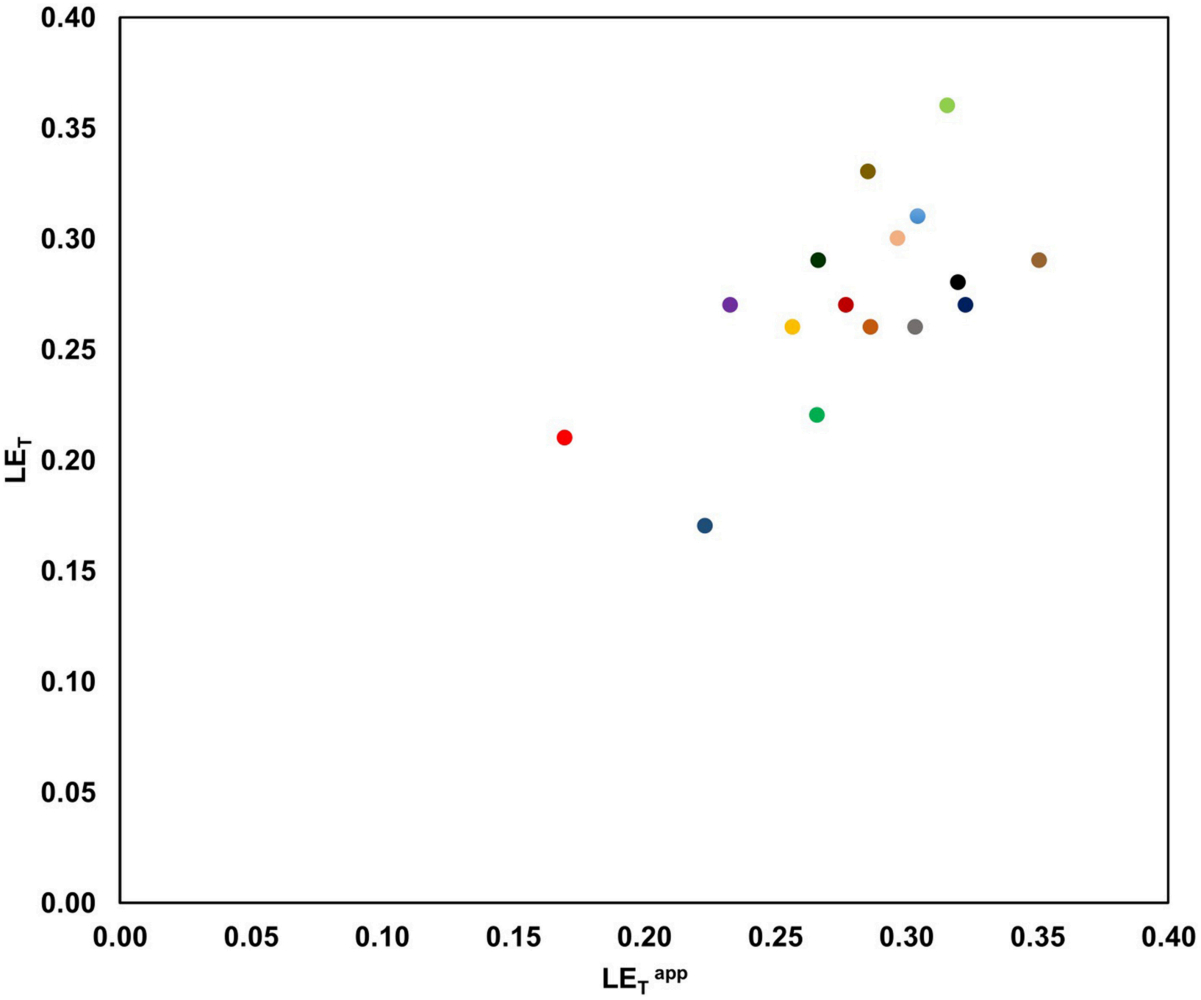
Preliminary comparison between ${\text{LE}}_{\text{T}}^{\text{app}}$ and LE_T_ for a set of 16 drug-sized literature compounds developed during FBDD studies. All compounds were elaborated using a linking strategy (on two fragments) for 10 different protein targets [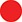/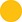
LDHA 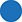
Replication Protein A (RPA) 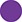/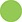
Blood coagulation factor Xa 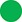
Bcl-2 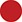
DOT1L 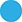
Hsp90 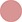/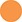
Pantothenate synthetase (PtS) 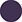
Blood coagulation factor XIa 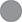
CK2 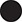
BACE1 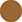
Endothiapepsin (Epn) 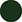
Bcl-x_L_ 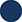
PKM2].

In addition, and according to our preliminary results, we found that ${\text{LE}}_{\text{T}}^{\text{app}}$ values predicted by this model were consistent with the LE_T_ values calculated experimentally from a set of 16 drug-sized molecules taken from scientific literature (Figure 2, Table 1S).

## Discussion

The present study pretends to propose the RGC model as an innovative and effective approach to apply in drug design. This model and, especially, the fragment “rescue” effect that is conceptually implicit, offer an alternative for the long-standing FBDD paradigm of designing compounds merely based on the intrinsic binding energy of fragments, facilitating the introduction of other decision–making criteria that are becoming increasingly common.

If the principles of the RGC model are considered together, it is possible to elucidate two major advantages. First, we count on a limited amount of data and in order to more clearly reveal any trends, ${\text{LE}}_{\text{T}}^{\text{app}}$ appears to increase as much as the LE_T_, and there appears to be no dramatic shift toward higher efficiencies for particular fragments or protein targets. Secondly, since a particular fragment could be directly rejected early in the process and there are many fragments by pocket in a typical FBDD campaign, this model might dramatically reduce the computational and synthetic costs, respectively (which is especially true in cases of three or more pockets).

The RGC model is, however, not free of inherent shortcomings. As a LE-derived metric, all fragments are assumed to maintain equal orientations both individually and as part of a larger chemical compound (Zartler and Shapiro, 2008), and phenomena such as hot spots (Zerbe et al., 2012; Rathi et al., 2017) or synergy (also called “super-additivity”) (Hebeisen et al., 2008; Nazaré et al., 2012) are not directly considered. Likewise, because its average-based nature, ΔG of each fragment is normalized not only at the number of non-hydrogen atoms but also on the number of component fragments. Therefore, the less accurate (or more extreme) ΔG values for fragments in each position are, the greater the difference expected between the ${\text{LE}}_{\text{T}}^{\text{app}}$ and the LE_T_. However, we believe that the impact of these hurdles could be minimized, improving the confidence of any potential fragment “rescue,” if additional energy terms derived from rigid body barrier (ΔG_rigid_), linker binding (ΔG_binding_) or strain (ΔG_strain_) (Murray and Verdonk, 2006; Cherry and Mitchell, 2008) are included and the ΔG values are both accurate and above a reasonable cut-off value.

A final examination about the implications of this work leads us to assert that the fragment “rescue” phenomenon is far from being new: it has already occurred and continues to occur, but usually during a lead optimization instead an earlier hit discovery phase. The rationale for this statement lies in two central facts. First, we currently know that the LE value of a compound tends to decrease during optimization process (Bembenek et al., 2009), while both its lipophilicity and its MW (and, hence, the number of non-hydrogen atoms -N) tends to increase (Ferenczy and Keseru, 2016). Second, the energy of supramolecular interactions is widely known to be largely different depending on the chemical moiety involved and, thus, ionic and hydrogen bonds are expected to account for a larger part of the drug-receptor binding energy (i.e., its ΔG) compared with hydrophobic interactions (Ermondi and Caron, 2006). Therefore, it is decidedly inviting to believe that, in many drug discovery initiatives, both the increase in LE and the decrease in MW and lipophilicity observed during lead optimization-could be explained by the addition of large and hydrophobic chemical moieties such as those cyclic aliphatic, which have a much smaller ΔG/N ratio compared to other fragments. Interestingly, these aliphatic moieties have been recently suggested by some authors to be more “developable” compared its aromatic analogs, which could be an additional factor behind this phenomenon (Lovering et al., 2009).

The RGC model presented in this study is based on the assumption that the LE of a drug-sized molecule may be estimated using the relative contribution of each component fragment. We believe this model could serve as a complementary benchmark for medicinal chemists in experimental or virtual fragment-based screening campaigns. Likewise, we consider that the RGC model could be implemented with other metrics based on either LE or a potency/size ratio and could be eventually adjusted to consider not only “linking” but also “growing” or “merging” as alternative fragment elaboration strategies.

## Data Availability

The raw data supporting the conclusions of this manuscript will be made available by the authors, without undue reservation, to any qualified researcher.

## Author Contributions

AV planned and performed the entire *in silico* work presented in this study, contributed to the analysis and interpretation of data, and assisted the writing, editing, and submission of this manuscript. AG made substantial contributions to the data analysis, critical revision for important intellectual content, and document editing. All authors have read and approved the final manuscript.

### Conflict of Interest Statement

The authors declare that the research was conducted in the absence of any commercial or financial relationships that could be construed as a potential conflict of interest.
