# Supplementary material for: Pushing the Ligand Efficiency Metrics: Relative Group Contribution (RGC) Model as a Helpful Strategy to Promote a Fragment “Rescue” Effect
Source: Front Chem. 2019 Aug 16;7:564. doi: 10.3389/fchem.2019.00564 (PMC6710606; doi:10.3389/fchem.2019.00564)
Supplement: Supplementary file 1 [file Table_1.DOCX]

C_25_H_31_N_3_O

| **Molecule** | **Target** | **IC_50_/K_D_/K_i_ (μm)** | **LE_T_** | **LE_Tapp_** | **Ref.** |
| --- | --- | --- | --- | --- | --- |
| 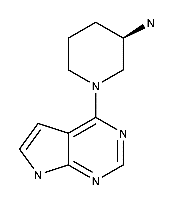  C_11_H_15_N_5_ | Hystone methyltransferase  DOT1L | 240 | 0.31 |  | (Möbitz et al., 2017) |
| 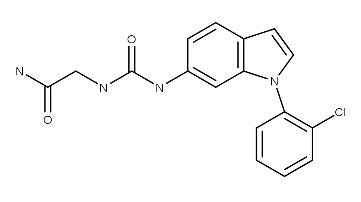  C_17_H_15_ClN_4_O_2_ | Hystone methyltransferase  DOT1L | 9 | 0.29 |  | (Möbitz et al., 2017) |
| 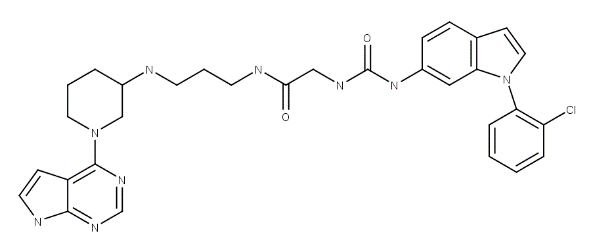 | Hystone methyltransferase  DOT1L | 0.004 | 0.27 | 0.27 | (Möbitz et al., 2017) |
| 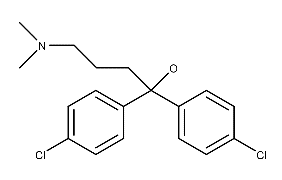  C_18_H_21_Cl_2_NO | BCL-2 | 20 | 0.29 |  | (Petros et al., 2010) |
| 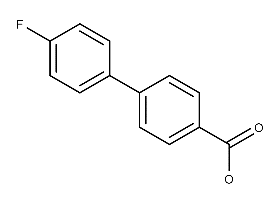  C_31_H_34_ClN_9_O_2_ | BCL-2 | 300 | 0.30 |  | (Petros et al., 2010) |
| 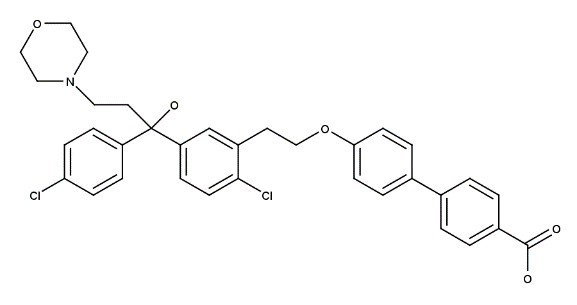  C_13_H_9_FO_2_ | BCL-2 | 0.22 | 0.22 | 0.26 | (Petros et al., 2010) |
| 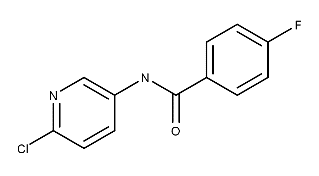  C_12_H_8_ClFN_2_O  C_35_H_35_Cl_2_NO_5_ | BACE-1 | 114 | 0.32 |  | (Jordan et al., 2016) |
| 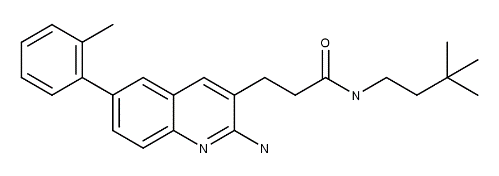 | BACE-1 | 0.14 | 0.32 |  | (Jordan et al., 2016) |
| 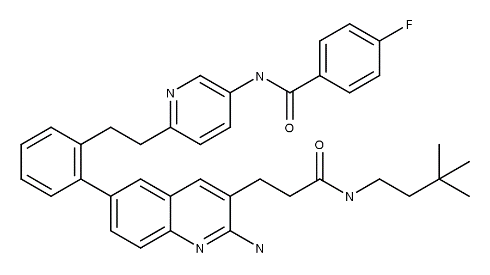  C_38_H_40_FN_5_O_2_ | BACE-1 | 0.0004 | 0.28 | 0.32 | (Jordan et al., 2016) |
| 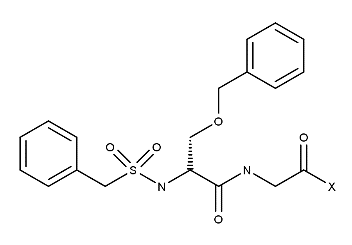  C_20_H_23_XN_2_O_4_S | Factor Xa | 5500 | 0.11 |  | (Jaegle et al., 2017) |
| 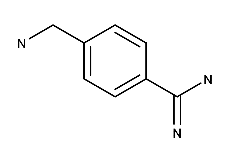  C_8_H_11_N_3_ | Factor Xa | 680 | 0.39 |  | (Jaegle et al., 2017) |
| 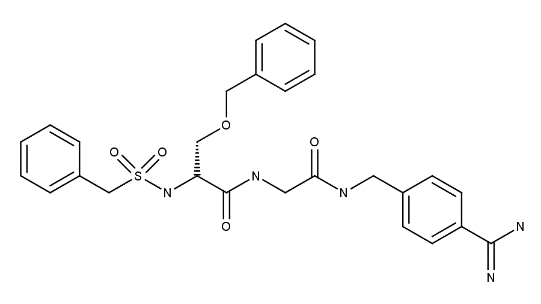  C_28_H_33_N_5_O_4_S | Factor Xa | 0.029 | 0.27 | 0.23 | (Jaegle et al., 2017) |
| 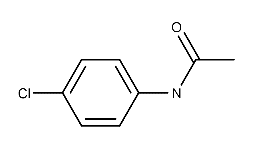  C_8_H_8_ClNO | Factor Xa | 326 | 0.43 |  | (Nazaré et al., 2012) |
| 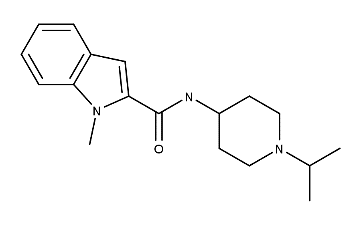  C_18_H_25_N_3_O | Factor Xa | 281 | 0.22 |  | (Nazaré et al., 2012) |
| 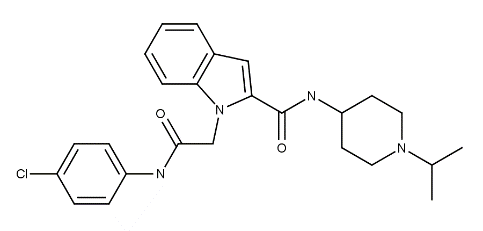  C_25_H_29_ClN_4_O_2_ | Factor Xa | 0.003 | 0.36 | 0.32 | (Nazaré et al., 2012) |
| 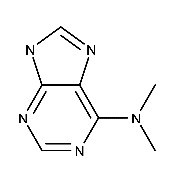  C_7_H_9_N_5_ | Hsp90 | 1500 | 0.32 |  | (Barker et al., 2010) |
| 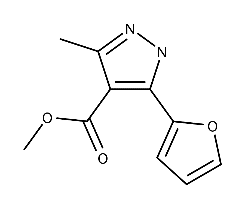  C_10_H_10_N_2_O_3_ | Hsp90 | 1000 | 0.27 |  | (Barker et al., 2010) |
| 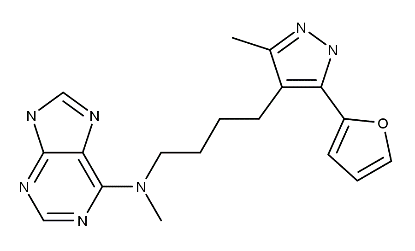  C_18_H_21_N_7_O | Hsp90 | 1.5 | 0.31 | 0.31 | (Barker et al., 2010) |
| 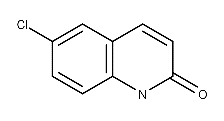  C_9_H_6_ClNO | Factor XIa | 70 | 0.47 |  | (Fjellström et al., 2015) |
| 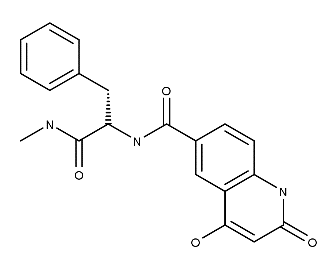  C_20_H_19_N_3_O_4_ | Factor XIa | 1400 | 0.14 |  | (Fjellström et al., 2015) |
| 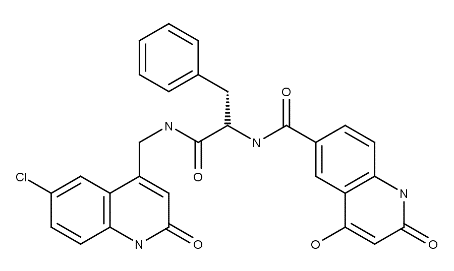  C_29_H_23_ClN_4_O_5_ | Factor XIa | 0.0005 | 0.33 | 0.29 | (Fjellström et al., 2015) |
| 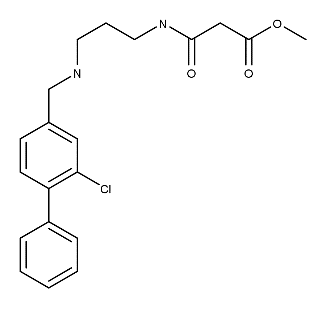  C_20_H_23_ClN_2_O_3_ | CK2 | 250 | 0.19 |  | (Brear et al., 2016) |
| 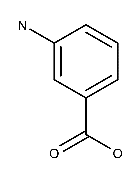  C_7_H_7_NO_2_ | CK2 | >500 | <0.45 |  | (Brear et al., 2016) |
| 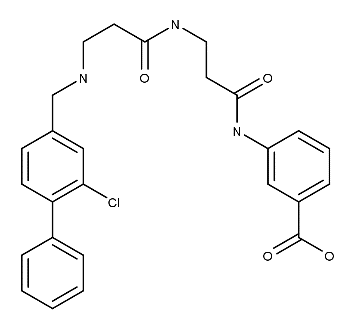  C_26_H_26_ClN_3_O_4_ | CK2 | 0.32 | 0.26 | 0.30 | (Brear et al., 2016) |
| 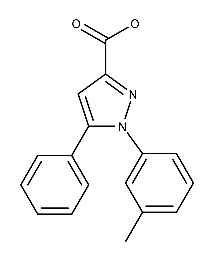  C_17_H_14_N_2_O_2_ | Replication protein A (RPA) | 580 | 0.21 |  | (Frank et al., 2013) |
| 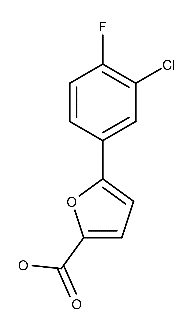  C_11_H_6_ClFO_3_ | Replication protein A (RPA) | 1400 | 0.24 |  | (Frank et al., 2013) |
| 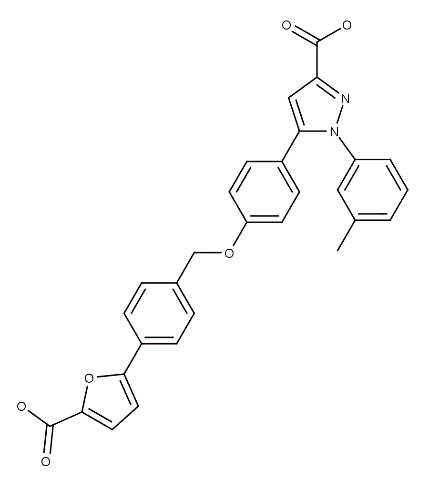  C_29_H_22_N_2_O_6_ | Replication protein A (RPA) | 20 | 0.17 | 0.22 | (Frank et al., 2013) |
| 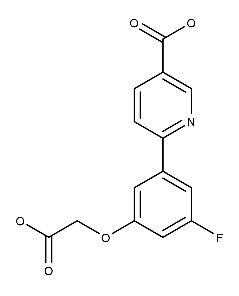  C_14_H_10_FNO_5_ | LDHA | 1300 | 0.19 |  | (Kohlmann et al., 2013) |
| 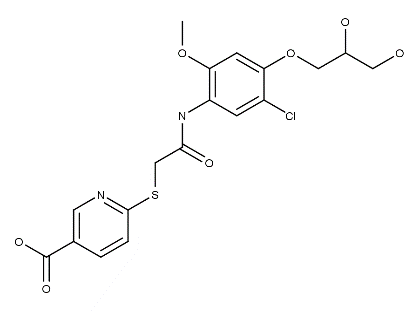  C_18_H_19_ClN_2_O_7_S | LDHA | 360 | 0.16 |  | (Kohlmann et al., 2013) |
| 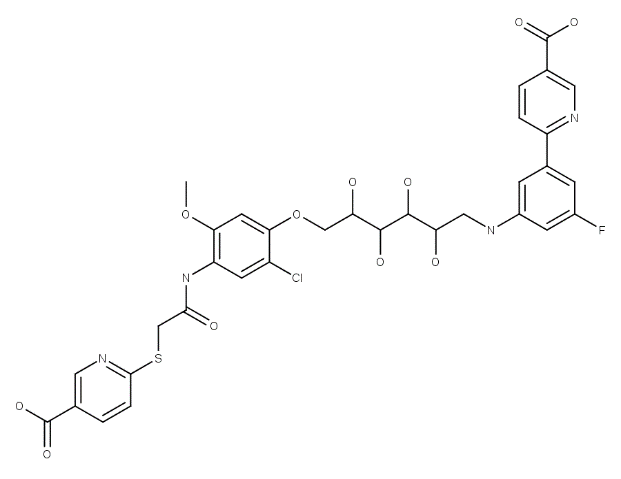  C_33_H_32_ClFN_4_O_11_S | LDHA | 0.019 | 0.21 | 0.17 | (Kohlmann et al., 2013) |
| 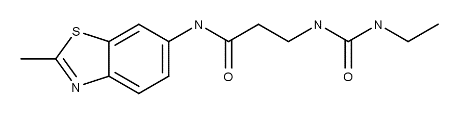  C_14_H_18_N_4_O_2_S | LDHA | 160 | 0.25 |  | (Ward et al., 2012) |
| 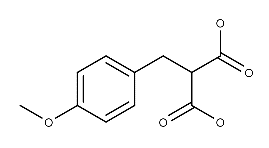  C_11_H_12_O_5_ | LDHA | 1100 | 0.25 |  | (Ward et al., 2012) |
| 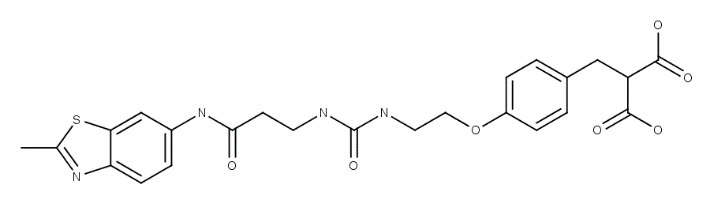  C_24_H_26_N_4_O_7_S | LDHA | 0.13 | 0.26 | 0.26 | (Ward et al., 2012) |
| 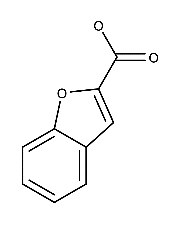  C_9_H_6_O_3_ | Pantothenate synthetase (PtS) | 1000 | 0.34 |  | (Sledz et al., 2010) |
| 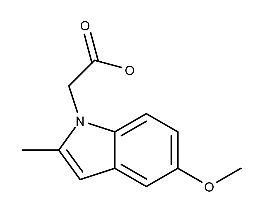  C_12_H_13_NO_3_ | Pantothenate synthetase (PtS) | 800 | 0.26 |  | (Sledz et al., 2010) |
| 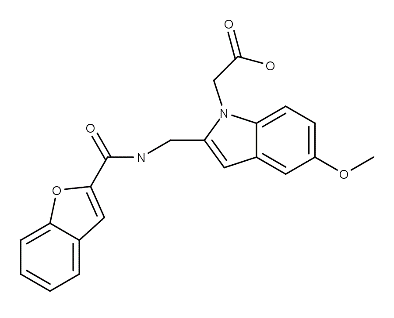  C_21_H_18_N_2_O_5_ | Pantothenate synthetase (PtS) | 0.86 | 0.30 | 0.30 | (Sledz et al., 2010) |
| 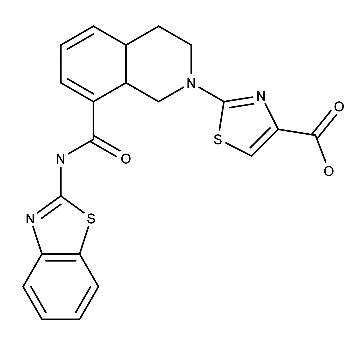  C_21_H_18_N_4_O_3_S_2_ | Bcl-x_L_ | 0.091 | 0.32 |  | (Tao et al., 2014) |
| 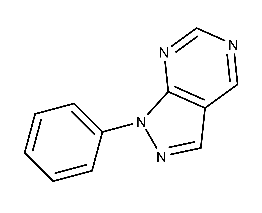  C_11_H_8_N_4_ | Bcl-x_L_ | 4000 | 0.22 |  | (Tao et al., 2014) |
| 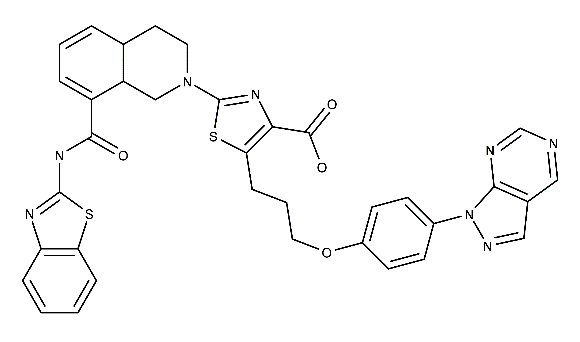  C_35_H_30_N_8_O_4_S_2_ | Bcl-x_L_ | 0.000042 | 0.29 | 0.27 | (Tao et al., 2014) |
| 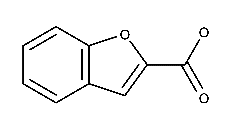  C_9_H_6_O_3_ | Pantothenate synthetase (PtS) | 1000 | 0.34 |  | (Hung et al., 2009) |
| 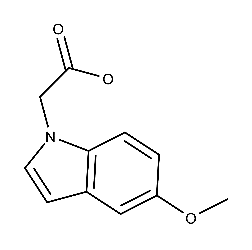  C_11_H_11_NO_3_ | Pantothenate synthetase (PtS) | 500 | 0.30 |  | (Hung et al., 2009) |
| 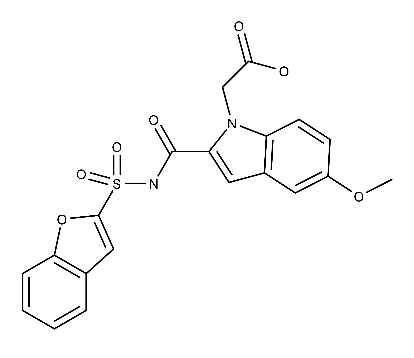  C_20_H_16_N_2_O_7_S | Pantothenate synthetase (PtS) | 1.8 | 0.26 | 0.29 | (Hung et al., 2009) |
| 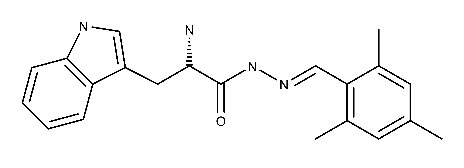  C_21_H_24_N_4_O | Endothiapepsin (Epn) | 12.8 | 0.27 |  | (Mondal et al., 2016) |
| 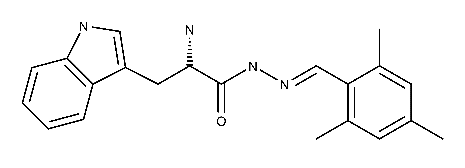  C_21_H_24_N_4_O | Endothiapepsin (Epn) | 12.8 | 0.27 |  | (Mondal et al., 2016) |
| 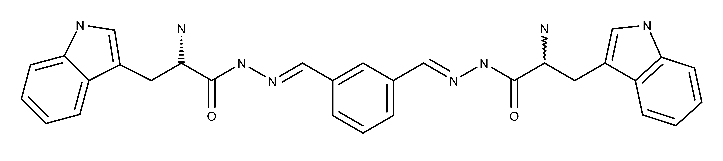  C_30_H_30_N_8_O_2_ | Endothiapepsin (Epn) | 0.054 | 0.29 | 0.35 | (Mondal et al., 2016) |
| 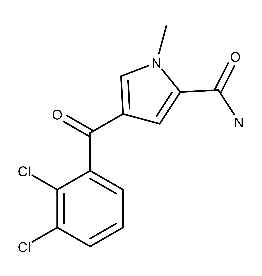  C_13_H_10_Cl_2_N_2_O_2_ | Pyruvate kinase M2 isoform (PKM2) | 17 | 0.34 |  | (Matsui et al., 2017) |
| 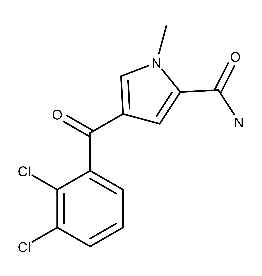  C_13_H_10_Cl_2_N_2_O_2_ | Pyruvate kinase M2 isoform (PKM2) | 17 | 0.34 |  | (Matsui et al., 2017) |
| 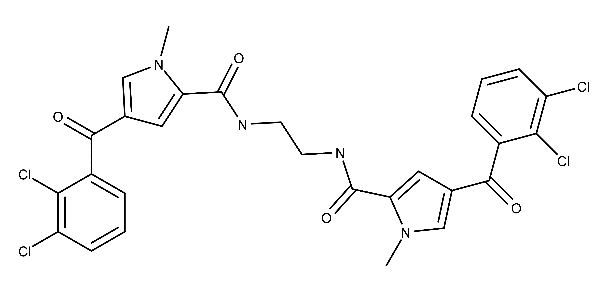  C_28_H_22_Cl_4_N_4_O_4_ | Pyruvate kinase M2 isoform (PKM2) | 0.014 | 0.27 | 0.32 | (Matsui et al., 2017) |

**Table 1S. Starting fragments and their optimized drug-sized compound (after fragment linking) for a set of different protein targets.** LE_T_^app^ was calculated for each drug-sized compound and adjusted by the number of non-hydrogen atoms if necessary.

**References**

Barker, J. J., Barker, O., Courtney, S. M., Gardiner, M., Hesterkamp, T., Ichihara, O., et al. (2010). Discovery of a novel Hsp90 inhibitor by fragment linking. *ChemMedChem* 5, 1697–1700. doi:10.1002/cmdc.201000219.

Brear, P., De Fusco, C., Hadje Georgiou, K., Francis-Newton, N. J., Stubbs, C. J., Sore, H. F., et al. (2016). Specific inhibition of CK2α from an anchor outside the active site. *Chem. Sci.* 7, 6839–6845. doi:10.1039/c6sc02335e.

Fjellström, O., Akkaya, S., Beisel, H. G., Eriksson, P. O., Erixon, K., Gustafsson, D., et al. (2015). Creating novel activated factor XI inhibitors through fragment based lead generation and structure aided drug design. *PLoS One* 10. doi:10.1371/journal.pone.0113705.

Frank, A. O., Feldkamp, M. D., Kennedy, J. P., Waterson, A. G., Pelz, N. F., Patrone, J. D., et al. (2013). Discovery of a potent inhibitor of replication protein A protein-protein interactions using a fragment-linking approach. *J. Med. Chem.* 56, 9242–9250. doi:10.1021/jm401333u.

Howard, N., Abell, C., Blakemore, W., Chessari, G., Congreve, M., Howard, S., et al. (2006). Application of Fragment Screening and Fragment Linking to the Discovery of Novel Thrombin. *J. Med. Chem.* 49, 1346–1355.

Hung, A. W., Silvestre, H. L., Wen, S., Ciulli, A., Blundell, T. L., and Abell, C. (2009). Application of Fragment Growing and Fragment Linking to the Discovery of Inhibitors of Mycobacterium tuberculosis Pantothenate Synthetase. *Angew. Chemie Int. Ed. English* 48, 8452–8456. doi:10.1002/anie.200903821.

Jaegle, M., Steinmetzer, T., and Rademann, J. (2017). Protein-Templated Formation of an Inhibitor of the Blood Coagulation Factor Xa through a Background-Free Amidation Reaction. *Angew. Chemie - Int. Ed.* 56, 3718–3722. doi:10.1002/anie.201611547.

Jordan, J. B., Whittington, D. A., Bartberger, M. D., Sickmier, E. A., Chen, K., Cheng, Y., et al. (2016). Fragment-Linking Approach Using19F NMR Spectroscopy to Obtain Highly Potent and Selective Inhibitors of β-Secretase. *J. Med. Chem.* 59, 3732–3749. doi:10.1021/acs.jmedchem.5b01917.

Kohlmann, A., Zech, S. G., Li, F., Zhou, T., Squillace, R. M., Commodore, L., et al. (2013). Fragment growing and linking lead to novel nanomolar lactate dehydrogenase inhibitors. *J. Med. Chem.* 56, 1023–1040. doi:10.1021/jm3014844.

Matsui, Y., Yasumatsu, I., Asahi, T., Kitamura, T., Kanai, K., and Ubukata, O. (2017). Discovery and structure-guided fragment-linking as a pyruvate kinase M2 activator. *Bioorg. Med. Chem.* 25, 3540–3546. doi:10.1016/j.bmc.2017.05.004.

Möbitz, H., Machauer, R., Holzer, P., Vaupel, A., Stauffer, F., Ragot, C., et al. (2017). Discovery of Potent, Selective, and Structurally Novel Dot1L Inhibitors by a Fragment Linking Approach. *ACS Med. Chem. Lett.* 8, 338–343. doi:10.1021/acsmedchemlett.6b00519.

Mondal, M., Radeva, N., Fanlo-Virgós, H., Otto, S., Klebe, G., and Hirsch, A. K. H. (2016). Fragment Linking and Optimization of Inhibitors of the Aspartic Protease Endothiapepsin: Fragment-Based Drug Design Facilitated by Dynamic Combinatorial Chemistry. *Angew. Chem. Int. Ed. Engl.* 55, 9422–9426.

Nazaré, M., Matter, H., Will, D. W., Wagner, M., Urmann, M., Czech, J., et al. (2012). Fragment deconstruction of small, potent factor xa inhibitors: Exploring the superadditivity energetics of fragment linking in protein-ligand complexes. *Angew. Chemie - Int. Ed.* 51, 905–911.

Petros, A. M., Huth, J. R., Oost, T., Park, C. M., Ding, H., Wang, X., et al. (2010). Discovery of a potent and selective Bcl-2 inhibitor using SAR by NMR. *Bioorganic Med. Chem. Lett.* 20, 6587–6591. doi:10.1016/j.bmcl.2010.09.033.

Sledz, P., Silvestre, H. L., Hung, A. W., Ciulli, A., Blundell, T. L., and Abell, C. (2010). Optimization of the interligand Overhauser effect for fragment linking: application to inhibitor discovery against Mycobacterium tuberculosis pantothenate synthetase. *J Am Chem Soc* 132, 4544–5. doi:10.1002/path.1711620107.

Tao, Z., Hasvold, L., Wang, L., Wang, X., Petros, A. M., Park, C. H., et al. (2014). Discovery of a Potent and Selective BCL ‑ X. *ACS Med. Chem. Lett.* 5, 1088−1093.

Ward, R. A., Brassington, C., Breeze, A. L., Caputo, A., Critchlow, S., Davies, G., et al. (2012). Design and synthesis of novel lactate dehydrogenase a inhibitors by fragment-based lead generation. *J. Med. Chem.* 55, 3285–3306. doi:10.1021/jm201734r.
