# Supplementary material for: Pushing the Ligand Efficiency Metrics: Relative Group Contribution (RGC) Model as a Helpful Strategy to Promote a Fragment “Rescue” Effect
Source: Front Chem. 2019 Aug 16;7:564. doi: 10.3389/fchem.2019.00564 (PMC6710606; doi:10.3389/fchem.2019.00564)
Supplement: Supplementary file 2 [file Table_2.DOCX]

**TABLE OF MATHEMATICAL TERMS**

| $\boldsymbol{K}_{\boldsymbol{d}}$ | Equilibrium dissociation constant |
| --- | --- |
| $\boldsymbol{\Delta G}$ | Gibbs-free energy change |
| $\boldsymbol{\Delta\Delta G}$ | Difference between two distinct Gibbs-free energy changes |
| $\boldsymbol{N}$ | Number of non-hydrogen atoms |
| $\boldsymbol{\Delta N}$ | Difference in the number of non-hydrogen atoms |
| $\boldsymbol{GE}$ | Group efficiency |
| $\boldsymbol{LE}_{\boldsymbol{T}}$ | Actual ligand efficiency (total) of a drug-sized compound. Also understood simply by LE. |
| $\boldsymbol{LE}_{\boldsymbol{T}}^{\boldsymbol{app}}$ | Predicted ligand efficiency (total) according to the RGC model. Also understood as $\bar{{LE}_{q}}$, it is, the weighted root mean square (WRMS) of LE. |
| $\boldsymbol{x}$ | Number of fragments composing a drug-sized compound |
| $\boldsymbol{\Delta G}_{\boldsymbol{T}}$ | Gibbs-free energy change (total) of a drug-sized compound |
| $\boldsymbol{N}_{\boldsymbol{T}}$ | Number of non-hydrogen atoms (total) of a drug-sized compound |
| $\boldsymbol{LE}_{\boldsymbol{i}}$ | Ligand efficiency for ‘ideal’ fragments with equal LE value |
| $\boldsymbol{LE}_{\boldsymbol{j}}$ | Ligand efficiency for fragment which LE value is known |
| $\boldsymbol{LE}_{\boldsymbol{\delta}}$ | Minimum ligand efficiency for fragments with unknown value. It corresponds to the minimum LE for accepting a fragment in unknown positions. |
| $\boldsymbol{LE}_{\boldsymbol{u}}$ | Minimum ligand efficiency for the last fragment with unknown value |
| $\boldsymbol{w}_{\boldsymbol{i}}$ | Weight for ‘ideal’ fragments assuming that they all have the same LE value. This parameter is expressed as the number of atoms or the corresponding percentage of the entire molecule. |
| $\boldsymbol{w}_{\boldsymbol{j}}$ | Weight for fragments which LE is known. This parameter is expressed as the number of atoms or the corresponding percentage of the entire molecule. |
| $\boldsymbol{w}_{\boldsymbol{\delta}}$ | Weight for fragments with unknown LE value. This parameter is expressed as the number of atoms or the corresponding percentage of the entire molecule. |
